# Positive Effects of Neutrophil Elastase Inhibitor (Sivelestat) on Gut Microbiome and Metabolite Profiles of Septic Rats

**DOI:** 10.3389/fcimb.2022.818391

**Published:** 2022-03-15

**Authors:** Yali Sun, Xianfei Ding, Yuqing Cui, Hongyi Li, Dong Wang, Huoyan Liang, Shaohua Liu, Xiaojuan Zhang, Haixu Wang, Tongwen Sun

**Affiliations:** General ICU, The First Affiliated Hospital of Zhengzhou University, Henan Key Laboratory of Critical Care Medicine, Zhengzhou Key Laboratory of Sepsis, Henan Engineering Research Center for Critical Care Medicine, Zhengzhou, China

**Keywords:** Sivelestat, sepsis, neutrophil elastase inhibitor, gut microbiota, metabolomics

## Abstract

**Background:**

Neutrophil elastase (NE) is associated with sepsis occurrence and progression. We hypothesized that the NE inhibitor Sivelestat might modulate abnormal gut microbiota and metabolites during sepsis.

**Methods:**

Sixty Sprague-Dawley (SD) rats were randomly divided into sham control (SC), sepsis (CLP), and sepsis+Sivelestat (Sive) groups. The rats’ survival status was monitored for 24 hours postoperatively, and feces were collected for microbiome and non-targeted metabolomics analyses.

**Results:**

Sivelestat administration significantly improved the survival of septic rats (80% vs 50%, *P* = 0.047). Microbiome analysis showed that the microbiota composition of rats in the CLP group was significantly disturbed, as potential pathogens such as *Escherichia-Shigella* and *Gammaproteobacteria* became dominant, and the beneficial microbiota represented by *Lactobacillus* decreased. These changes were reversed in Sive group, and the overall microbial status was restored to a similar composition to SC group. Differential analysis identified 36 differential operational taxonomic units and 11 metabolites between the Sive and CLP groups, such as 6-Aminopenicillanic acid, gamma-Glutamyl-leucine, and cortisone (variable importance in projection>1and *P*<0.05). These discriminatory metabolites were highly correlated with each other and mainly involved in the phenylalanine, tyrosine, and tryptophan biosynthesis pathways. Integrated microbiome and metabolome analyses found that almost all Sivelestat-modulated microbes were associated with differential metabolites (*P* < 0.05), such as *Lactobacillu*s and some amino acids, suggesting that the Sivelestat-induced metabolic profile differences were in part due to its influence on the gut microbiome.

**Conclusion:**

Sivelestat administration in septic rats improved survival, gut microbiota composition and associated metabolites, which could provide new options for sepsis treatment.

## Background

Sepsis is a life-threatening organ dysfunction caused by a dysregulated host response to infection, which is associated with a high morbidity and mortality worldwide (Singer et al., 2016; Evans et al., 2021). Unfortunately, there is no specific anti-sepsis treatment (Cohen et al., 2015).

Previous studies have shown that neutrophils are key effectors of the host innate immune response. Neutrophil overstimulation or dysfunction is one of the major pathophysiological hallmarks of sepsis-associated acute lung injury (ALI) (Park et al., 2017; Aziz et al., 2018), which is the most frequent organ dysfunction caused by sepsis (Rubenfeld et al., 2005; Aziz et al., 2018). Neutrophils can interact with endothelial cells and release a range of cytokines, chemokines, and protein hydrolases, leading to unrestricted inflammation, lung dysfunction, and even death (Delgado-Rizo et al., 2017; Shen et al., 2017). Neutrophil elastase (NE), a neutrophil biomarker (Korkmaz et al., 2010), is the most important protease released by neutrophils. It is one of the major effectors in immune defense and inflammatory response regulation and associated with the occurrence and development of sepsis. NE can degrade the extracellular matrix, promote immune cell migration and induce the release of pro-inflammatory mediators, thereby inducing organ dysfunction (Groutas et al., 2011). Interestingly, NE can also degrade intestinal epithelial connexins, leading to changes in intestinal barrier permeability and intestinal flora imbalances. Animal experiments have shown that NE inhibition reduced the intestinal colonization of *Salmonella* and other pathogenic bacteria and prevented the occurrence of gastroenteritis (Gill et al., 2012). Therefore, we speculated that NE inhibitors could alleviate sepsis progression by alleviating inflammation, gut barrier dysfunction, and intestinal flora imbalance through a still unidentified mechanism.

Sivelestat is a highly specific NE inhibitor with beneficial effects in various conditions induced by acute inflammation and is currently the only drug approved for the treatment of ALI/ARDS worldwide (Kawabata et al., 2002; Sakashita et al., 2007; Takemasa et al., 2012). Sivelestat has been shown to improve sepsis-associated organ injury by inhibiting the activation of inflammatory modulators such as the serine/threonine kinase and pro-inflammatory mediators, including high mobility group box 1 (Hagiwara et al., 2008; Li et al., 2016). However, although NE was associated with intestinal microbes, the effects of Sivelestat on gut microflora and metabolites during sepsis had not been investigated. This study aimed to verify whether Sivelestat could have therapeutic effects on septic rats, and decipher its potential impact to sepsis from the perspective of gut microorganisms and metabolites.

## Materials and Methods

### Drugs, Instruments, and Reagents

Sivelestat (0.1 g, H20203093, valid until 05/2022, Shanghai Huilun Jiangsu Pharmaceutical Co., Ltd.), Vanquish Ultra High-Performance Liquid Chromatograph (Thermo Fisher Scientific), Q Executive HFX High-Resolution Mass Spectrometer (Thermo Fisher Scientific), ultrasonicator (PS-60AL, Shenzhen Redbone Electronics Co., Ltd.), methanol and acetonitrile (CNW Technologies), ammonium acetate (SIGMA-ALDRICH), ammonium hydroxide (Fisher Chemical), ddH_2_O (Watsons).

### Animals and Fecal Sample Collection

A total of 60 adult 6-8-weeks-old male Sprague-Dawley rats were purchased from Charles River (Beijing, China). Rats were housed under controlled light, temperature, and humidity conditions (12/12 h light/dark cycle, 20–22 ˚C, 50–60%) and allowed unrestricted access to food and water. After one week of adaptation to the environment, the rats were randomly divided into three groups (n=20/group): sham-operated control (SC) group, cecum ligation and puncture to induce sepsis (CLP) group, and intraperitoneal administration of Sivelestat (Sive) group. The experiments were carried out according to the guidelines established by the National Institutes of Health and approved by the Committee of Zhengzhou University.

CLP procedure was performed to induce sepsis based on previously published protocols (Rittirsch et al., 2009; Sun et al., 2020). In short, 10% chloral hydrate (350 mg/kg) was injected intraperitoneally (i.p) to anesthetize rats, then the abdomen was shaved and disinfected, and a 1-2-cm incision was made to expose the cecum. The lower and middle 1/3 of the cecum was tightly ligated and punctured (medium ligation, inducing mid-grade sepsis), 1–2 drops of feces were squeezed out, and the cecum was retracted into the abdominal cavity. The abdominal muscle layer and the skin were closed with sterile 5 + 0 and 3 + 0 surgical sutures, respectively. Rats in the Sive group were injected i.p. with Sivelestat (50 mg/kg) 1 hour after CLP at a dose determined by previous studies (Hagiwara et al., 2008; Li et al., 2016) and our preliminary survival results. Rats in the CLP group were injected with the same volume of physiological saline as controls. The SC group was not ligated, and the cecum was not perforated, and the rest of the steps were consistent with the CLP group. All rats were injected subcutaneously with 10 mL of pre-warmed saline for fluid resuscitation and were placed under a warm light after surgery. In order to exclude the impact of antibiotic administration on intestinal flora, we did not give antibiotic treatment after operation. Finally, since the mortality of rats with mid-grade sepsis changed little more than 24 hours after modelling (Rittirsch et al., 2009), we observed the survival status of each group at 24 hours after surgery, and feces in the colon from surviving rats were collected and placed in two 2mL tubes, numbered and labeled, and immediately stored at -80°C for further testing.

### Fecal DNA Extraction and PCR Amplification

The sodium dodecyl sulfate (SDS) method was used to extract genomic DNA from samples, and then the DNA was diluted to 1 ng/μL with sterile water. The V3 and V4 regions were selected as sequencing regions, and the primer sequences for PCR amplification were 341F: CCTAYGGGRBGCASCAG; 806R: GGACTACNNGGGTATCTAAT. PCR products were quantified by enzyme-labeled methods and mixed in equal amounts according to PCR product concentrations. Then, PCR products were resolved with 2% agarose gel electrophoresis.

### 16S rRNA Sequencing

The library was constructed using the TruSeq^®^ DNA PCR-Free Sample Preparation Kit and quantified by Qubit and qPCR. The Illumina NovaSeq6000 platform was used for paired_end sequencing.

### 16S rRNA Sequencing Data Analysis

Raw Tags were obtained by truncating the Barcode and primer sequences from the raw downstream data, and FLASH(V1.2.7)was used to splice the sample reads (Magoč and Salzberg, 2011). The Tags quality control process of Qiime (V1.9.1) (Caporaso et al., 2010), and a series of strict filtering processes including tags interception, length filtering, chimera sequence removal (Rognes et al., 2016) were performed on the tags to obtain Effective Tags (Bokulich et al., 2013).

All sample Effective Tags were clustered OTUs (Operational Taxonomic Units) with 97% identity confirmed using Uparse (v7.0.1001) (Haas et al., 2011). Species taxonomic analysis of OTUs was performed using the Mothur method and the SSUrRNA database of SILVA138 (threshold set to 0.8–1) (Wang et al., 2007; Edgar, 2013). Phylogenetic relationships of OTUs were determined using MUSCLE software (Version 3.8.31) (Quast et al., 2013). Then, the data were homogenized and analyzed for diversity. Qiime software was used to calculate the alpha diversity index including observed-otus, Chao1, Shannon, Simpson, ace, Goods-coverage and PD_whole_tree. R software (version 4.0.5) was used to analyze the difference between groups of alpha diversity index and plot Rarefaction curve, rank abundance curve and species accumulation boxplot. Principal Co-ordinates Analysis (PCoA) and Unweighted Pair-group Method with Arithmetic Mean clustering tree (UPGMA) based on unifrac distance were used to present beta diversity and performed using WGCNA, stats, and R software. LEfSe software was used to perform LEfSe analysis. Other diagrams were generated using the R package (Logue et al., 2016; Walters et al., 2016).

### Fecal Metabolite Extraction

For each sample, 500 μL of extract solution (methanol:acetonitrile:water [2:2:1, v/v]), containing isotopically labeled internal standard mixture) was added to 25 mg of fecal material. Then, samples were mixed and incubated at -40°C for one hour. Afterward, the supernatant was collected by centrifugation (4°C, 12000 rpm, 15 min) for subsequent assays.

### Non-Targeted Metabolomics Data Acquisition

Chromatographic separation of the target compounds was performed using the Vanquish Ultra High-Performance Liquid Chromatograph with a Waters ACQUITY UPLC BEH Amide column (2.1 mm × 100 mm, 1.7 μm). A phase of liquid chromatography was an aqueous phase containing 25 mmol/L ammonium acetate and 25 mmol/L ammonia, and B phase was acetonitrile (auto-sampler temperature: 4°C, injection volume: 2 μL). The primary and secondary mass spectrometry data acquisition was performed using the Thermo Q Exactive HFX mass spectrometer.

### Metabolomics Data Preprocessing and Annotation

ProteoWizard software was used to convert the raw data into mzXML format. Peak identification, peak extraction, peak alignment, and integration were performed using a self-written R package based on XCMS (Smith et al., 2006). The data were then matched against an in-house MS2 database (BiotreeDB, V2.1) for metabolite annotation (cutoff = 0.3).

### Multivariate Data Analysis and Identification of Potential Biomarkers

SIMCA software (V16.0.2) was used for logarithmic data conversion and centralization (CTR). Multivariate statistical analysis of the qualitative and quantitative metabolome results was performed by principal component analysis (PCA) and orthogonal projections to latent structures discriminant analysis (OPLS-DA), and the robustness of the OPLS-DA model was assessed by permutation tests. Subsequently, the differential metabolite markers were screened using variable importance in projection (VIP)>1 and *P*<0.05 as criteria. Then, we performed a hierarchical cluster analysis (HCA) of differential metabolites using the complete chain method and presented them as heatmaps.

### Statistical Analyses

Chi-square and Fisher’s exact tests were used for the analysis of differences in mortality between the experimental groups. Parametric and non-parametric tests were used to analyze between-group differences. T-tests and the Wilcoxon Mann-Whitney test were used for two-group comparisons. Analysis of variance (ANOVA) and the Kruskal Wallis H test were used for three-group comparisons, and the Tukey method was used for subsequent multiple comparisons. Data shown were mean ± SD or median (IQR). Alpha and beta diversity indices were calculated by Qiime (Version 1.9.1). The screening value of LDA score of LEfSe analysis was set to 4.The relative abundance of differential OTUs were z-transformed by R software and analyzed using the Mann-Whitney U test. Pearson and Spearman correlation analyses were used to calculate the correlations between metabolites or between flora and metabolites. *P* < 0.05 was considered statistically significant. All data were analyzed using SPSS 25.0, GraphPad Prism 8, and R software.

## Results

### Sivelestat Improved 24-h Survival in Septic Rats

**Table 1** shows the survival of the experimental groups of rats at 24 h after surgery. None of the rats in the SC group died. In contrast, the mortality rate in the CLP group was 50%, and the rats exhibited clinical signs of sepsis such as chills, weakness, reduced muscle activity, and cold extremities, indicating that the sepsis model was appropriate. Compared with the CLP group, the mortality rate in Sive group was only 20%, showing that Sivelestat had a significant therapeutic effect in septic rats (*P* = 0.047).

**Table 1:** Twenty-four-hour mortality of animals in each experimental group.

Group	Fatality rate (deaths/total)	P value
SC	0% (0/20)	0.001^a^
CLP	50% (10/20)	0.047^b^
Sive	20% (4/20)	

a Compared with CLP group.

b Compared with Sive group.

### Gut Microbial Profile Alterations in Rats

**Figure 1:**
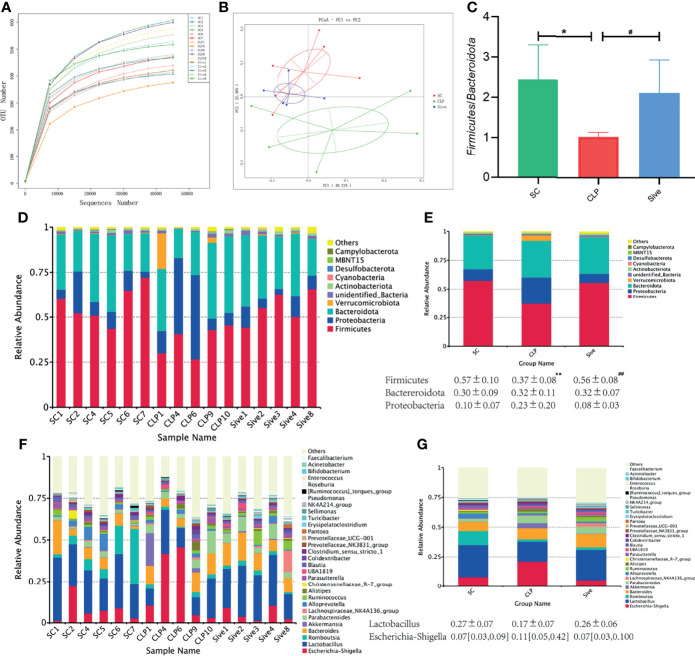
Effects of Sivelestat on gut microbiome diversity and structure. **(A)** Rarefaction Curve of samples showing the appropriateness of sequencing depth. **(B)** PCoA plot based on weighted unifrac distance of OTUs (97% similarity level). **(C)** The *Firmicutes*/*Bacteroidetes* ratios of the experimental groups. **(D, E)** Relative bacterial distribution at the phylum level. **(F, G)** Relative bacterial distribution at the genus level. ^*^Compared with the SC group; **^#^**compared with the CLP group; ^*^^,^**^#^***P*<0.05;^**^^,^**^##^***P*<0.014.

To investigate whether Sivelestat exerted its therapeutic effect by affecting the gut microbial community, we first performed alpha and beta diversity analysis of the fecal flora. **Supplementary Table 1** shows the index of alpha diversity in each group at a 97% concordance threshold. The observed_species, Shannon, chao1, and ACE index were higher in the CLP group compared to the SC group and showed a decreasing trend after Sivelestat treatment, but the difference was not statistically significant. Rarefaction and rank abundance curves were used to initially assess differences in the richness and diversity of microbial communities within samples and to establish whether the amount of sequencing data was appropriate (Li et al., 2013). The results showed that the number of sequences we obtained was adequate and plausible (**Figure 1A** and **Supplementary Figure 1A** **)**. In addition, a species accumulation boxplot showed that the sample was sufficient (**Supplementary Figure 1B**).

Then, PCoA analysis and a UPGMA clustering tree based on weighted unifrac distances were used to compare the beta diversity of groups. **Figure 1B** showed that the samples from the SC and CLP groups were farther apart, while the Sive group was closer to the SC group, and the differences were statistically significant (**Supplementary Figure 1C**). This suggested that sepsis caused significant changes in gut microbiome structure compared to that in healthy rats and there was a tendency to restore the abnormal gut microbiome after Sivelestat administration. Consistent with the PCoA results, the UPGMA clustering tree showed that the SC and Sive groups were clustered together, while the CLP group was separated from these two groups (**Supplementary Figure 1D**). In conclusion, these findings provided evidence for the therapeutic effects of Sivelestat through regulation of the abnormal gut microbial composition caused by sepsis.

### Dominant Bacteria Abundance Analysis

The relative abundance of dominant species at the phylum and genus levels was assessed to determine the effect of Sivelestat on gut bacterial abundance. **Figures 1D, E** showed that the dominant phyla in all three groups were *Firmicutes*, *Bacteroidota*, and *Proteobacteria*, which together accounted for more than 90% of the total. In contrast, the abundance of *Firmicutes* was significantly lower (*P* = 0.006), and the abundance of *Proteobacteria* was higher in the CLP group than in the SC group, indicating that the composition of the gut flora of septic rats was significantly disturbed. Interestingly, these changes were restored in the Sive group to levels similar to those in the SC group. We also found that the *Firmicutes/Bacteroidota* ratio was significantly decreased in the CLP group, while the SC and Sive groups had similar *Firmicutes/Bacteroidota* ratios (*P*<0.05, **Figure 1C**). The dominant bacteria at the genus level in each group of samples are shown in **Figures 1F, G** and **Supplementary Figure 2**. The most abundant genus in the phylum *Firmicutes* was *Lactobacillus*, and its abundance decreased in the CLP group, whereas the SC and Sive groups had similar *Lactobacillus* levels. Further, *Escherichia-Shigella* of the phylum *Proteobacteria* were enriched in the CLP group, but after Sivelestat treatment, levels were reduced and similar to those in the SC group. Overall, the results suggested that Sivelestat could potentially restore the abnormal gut bacterial composition in septic rats.

### Differential Bacterial Analysis

**Figure 2:**
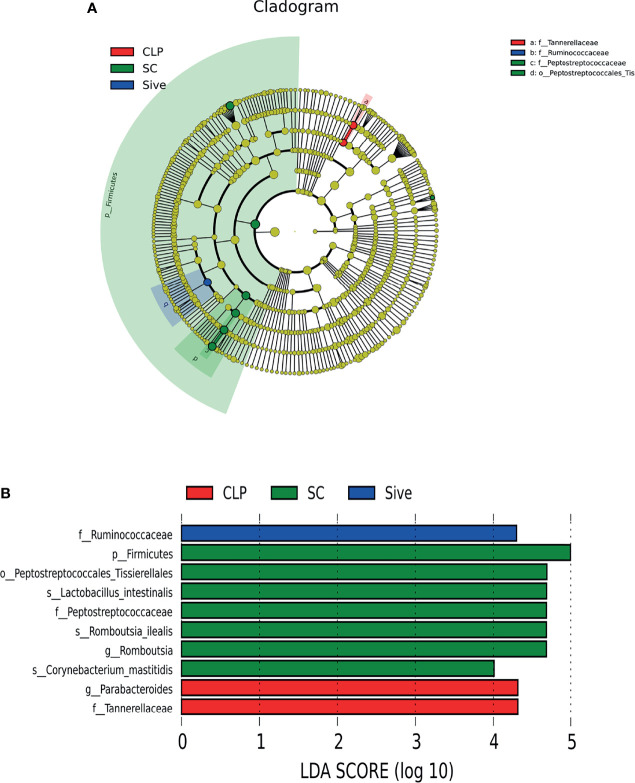
Linear discriminant analysis (LDA) integrated with effect size (LEfSe). **(A)** Taxonomic cladogram showing the phylogenetic distribution of the microbiota among experimental groups. **(B)** Bacteria with significant between-group differences (LDA score>4).

**Figure 3:**
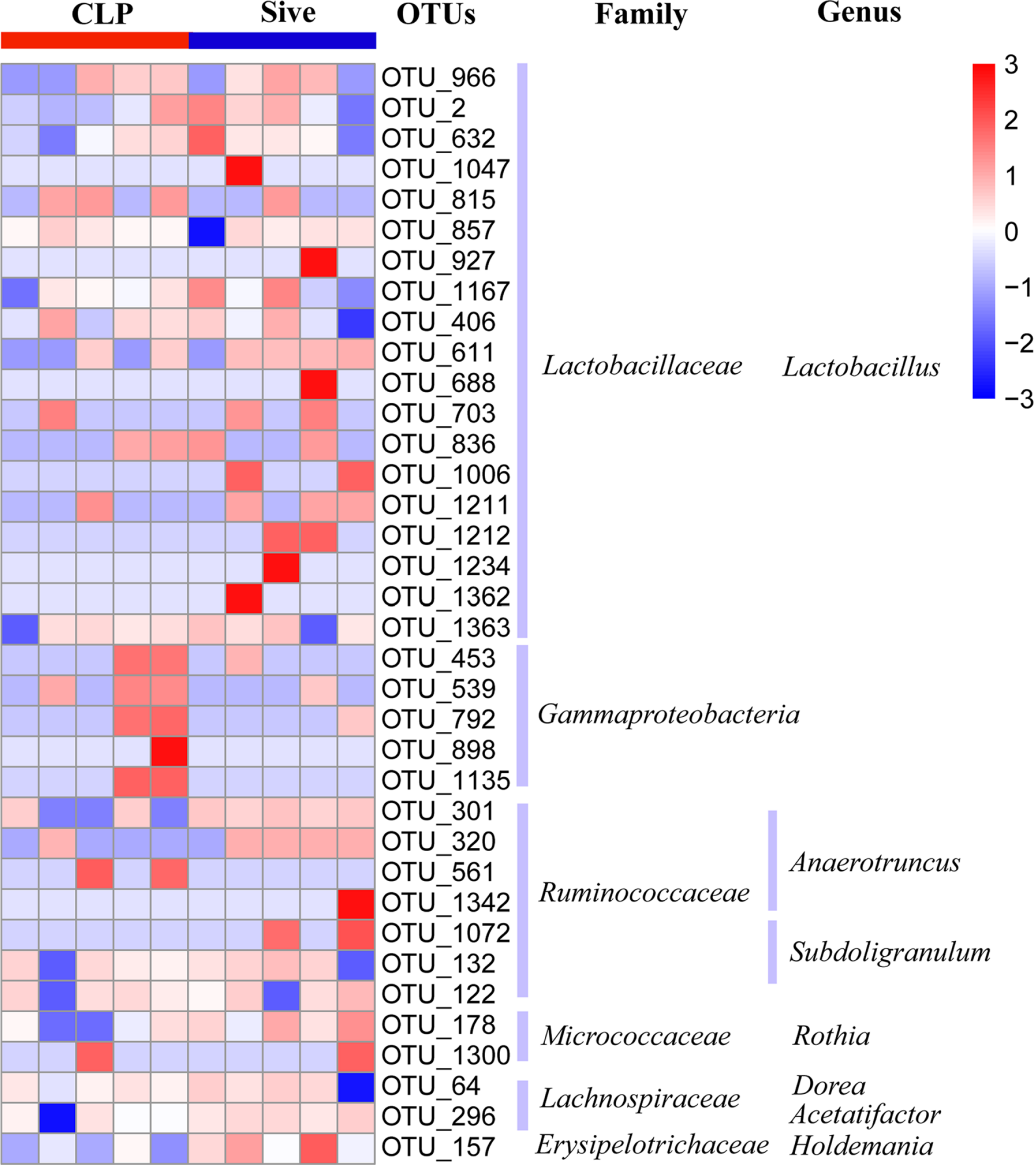
Relative abundance heatmap of 36 differential OTUs between the CLP and Sive groups. Data were z-transformed and analyzed using the Mann-Whitney U test. All 36 OTUs were classified into families and genera.

To determine the biological relevance of bacterial phyla, orders, families, genus and species, LEfSe was performed to identify the specific bacterial taxa in each experimental group. The cladogram showed that the taxa of the CLP group differed the most from the other two groups (**Figure 2A**). Sivelestat administration significantly increased the relative abundance of *Ruminococcaceae* and reduced the relative abundance of *Parabacteroides* and *Tannerellaceae* in septic rats (**Figure 2B** **)**. In addition, to further identify the key bacteria regulated by Sivelestat, we performed statistical tests on OTUs in the Sive and CLP groups and identified 36 differential OTUs. **Figure 3** shows the relative abundance and taxonomic information of the 36 OTUs. Sivelestat treatment increased the abundance of *Lactobacillus* and *Ruminococcaceae*, while the levels of potential pathogens such as *Gammaproteobacteria* were significantly reduced. These data suggested that Sivelestat could provide a protective effect by increasing beneficial bacteria and decreasing pathogenic bacteria during sepsis.

### Metabolic Profile Alterations in Septic Rats

**Figure 4:**
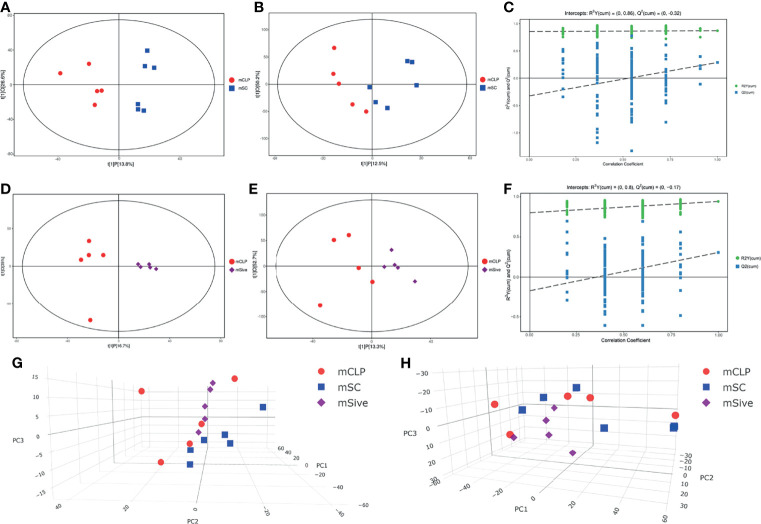
**(A–C)** Score scatter plot and Permutation test of the OPLS-DA model for the CLP and SC groups. **(D–F)** Score scatter plot and Permutation test of the OPLS-DA model for the Sive and CLP groups. **(E, F)** PCA scores of experimental groups. **(A, C, D, F, G)** Negative ion mode. **(B, E, H)** Positive ion mode.

Given that Sivelestat could modulate the abnormal microbiota of septic rats, we hypothesized that Sivelestat-regulated gut microbiota might affect the activity of certain metabolites and pathways, leading to beneficial effects. Therefore, a non-targeted metabolomics assay was performed to determine fecal metabolite profiles. Metabolites were detected using negative ion mode (NEG) and positive ion mode (POS) ionization methods. The total ion flow patterns of the experimental groups are shown in **Supplementary Figure 3**. The peak type of the CLP group was significantly different than that of the SC group, whereas the SC and Sive groups had similar peak types. Principal component analysis (PCA) and orthogonal projections to latent structures discriminant analysis (OPLS-DA) were carried out to visualize the overall metabolite distribution characteristics in the three experimental groups and identify significantly altered metabolites. The results showed that the CLP group was clearly distinguishable from the SC group, while the Sive group diverged from the CLP group and showed similarities to the SC group, indicating that Sivelestat restored the abnormal metabolic profile of septic rats to a state similar to that of healthy rats. We also performed an additional robustness assessment of the OPLS-DA model, and the results showed that the model, particularly the negative ion mode, could reliably identify between-group differences (**Figure 4**).

### Differential Metabolite and Pathway Identification

**Figure 5:**
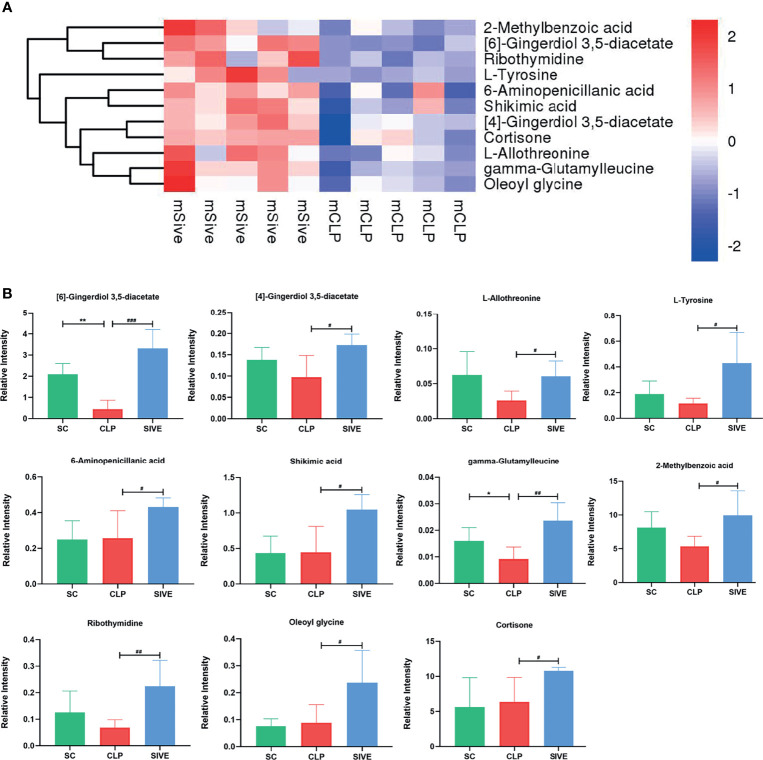
Differential metabolites between the Sive and CLP groups. **(A)** Heatmap of hierarchical clustering analysis based on metabolite z-normalized abundances. **(B)** Relative intensities of metabolites in the experimental groups. ^*^Compared with the SC group; **^#^**compared with the CLP group; ^*^^,^**^#^***P*<0.05; ^**^^,^**^##^***P*<0.01; **^###^***P*<0.001.

**Figure 6:**
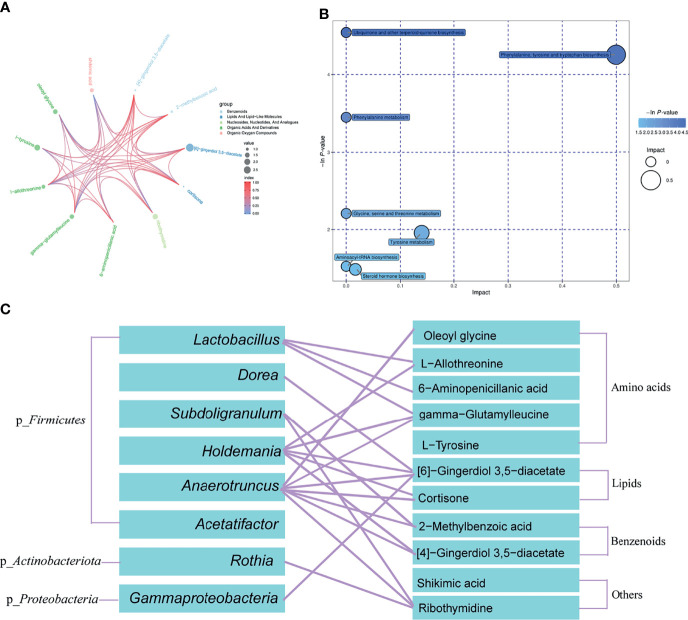
Correlation Analysis and Differential Metabolic Pathway enrichment. **(A)** Chord analysis of differential metabolites between the Sive and CLP groups was used to visualize the relationship between substance classification and content variation. **(B)** Pathway enrichment and statistical significance of differential metabolites between the Sive and CLP groups. **(C)** Correlation analysis of differential bacterial genera and metabolites between the Sive and CLP groups.

To identify the main metabolites and pathways affected by Sivelestat, the VIP values of the first principal component in the OLPS-DA model were obtained, and the metabolites with VIP>1 and *P*<0.05 were considered as differential metabolites. A total of 11 differential metabolites were identified, most of which were organic acids and derivatives (**Table 2**). A heatmap of the relative levels of the differential metabolites in each sample from the two groups is shown in **Figure 5A**. The levels of 2-methylbenzoic acid, [6]-gingerdiol 3,5-diacetate, ribothymidine, L-tyrosine, 6-aminopenicillanic acid, shikimic acid, [4]-gingerdiol 3,5-diacetate, cortisone, L-allothreonine, gamma-glutamylleucine, and oleoyl glycine were significantly higher in the Sive group compared to the CLP group. Upon examining the levels of these 11 metabolites in healthy rats, we found that [6]-gingerdiol 3,5-diacetate and gamma-glutamylleucine levels were significantly reduced in the CLP group compared to the SC group and that their levels increased after Sivelestat treatment (*P* < 0.05, **Figure 5B**). Moreover, [4]-gingerdiol 3,5-diacetate, L-allothreonine, L-tyrosine, 2-methylbenzoic acid, and ribothymidine levels were lowest in the CLP group and higher in the Sive and SC groups, although the differences were not statistically significant. **Figure 6A** and **Supplementary Figure 4A** show the results of correlation analysis of the differential metabolites, indicating a strong correlation between these 11 metabolites. In addition, pathway enrichment analysis was performed using the KEGG and MetaboAnalyst databases. Sivelestat treatment mainly affected phenylalanine, tyrosine, and tryptophan biosynthesis and tyrosine metabolism pathways (**Figure 6B**). In conclusion, our metabolomic analysis results suggested that Sivelestat could modulate specific metabolites and metabolic pathways in septic rats.

**Table 2:** Major differential metabolites between Sive and ClP groups.

MS2 name	Rt	mz	VIP	*P* value	FC(Sive/CLP)	FC(CLP/SC)	Super class
[6]-Gingerdiol 3,5-diacetate	129.823	379.2134	1.92	0.001	7.30	0.28	1
[4]-Gingerdiol 3,5-diacetate	116.863	351.1824	1.25	0.019	1.77	0.54	2
L-Allothreonine	378.976	118.0501	1.85	0.018	2.30	0.27	3
L-Tyrosine	324.667	180.0660	1.83	0.042	3.68	0.21	3
6-Aminopenicillanic acid	117.779	215.0500	1.76	0.043	1.68	1.03	3
Shikimic acid	151.809	173.0450	1.091	0.012	2.34	1.03	4
gamma-Glutamylleucine	405.695	259.1300	1.77	0.004	2.57	0.57	3
2-Methylbenzoic acid	117.067	135.0444	1.88	0.031	1.86	0.87	2
Ribothymidine	188.1215	257.0780	1.79	0.021	3.29	0.54	5
Oleoyl glycine	89.83385	338.2710	1.22	0.042	2.67	0.80	3
Cortisone	112.359	359.1904	1.38	0.046	1.69	1.14	1

Supper class: 1. Lipids and lipid-like molecules; 2. Benzenoids; 3. Organic acids and derivatives; 4. Organic oxygen compounds; 5. Nucleosides, nucleotides, and analogues.

### Microbial-Metabolite Association Analysis

Based on our microbiome and metabolomics data, we performed a combined microbial-metabolite analysis within each group. **Supplementary Figures 4B, C** shows the bacterial genera and metabolites with statistically significant correlations in the CLP and Sive groups. Interestingly, we found more microorganisms and metabolites with statistically significant correlations within the Sive group compared with the CLP group. In addition, we performed Pearson correlation analysis of differential genera and metabolites in the CLP and Sive groups. We found that all differential metabolites except shikimic acid were correlated with differential genera other than *Acetatifactor*, especially *Lactobacillus*, *Holdmania*, and *Anaerotruncus* of phylum *Firmicutes* (*P* < 0.05, **Figure 6C** and **Supplementary Figure 5**). These results suggested that the altered metabolites after Sivelestat administration could be attributed to its effect on the microbiome.

## Discussion

In this study, we characterized the beneficial effects of Sivelestat administration on the gut microbiome and metabolic profiles in septic rats. We found that 1) the microbiota composition of rats in the CLP group was significantly disturbed, as potential pathogens such as *Escherichia-Shigella* and *Gammaproteobacteria* became dominant, and beneficial microbiota represented by *Lactobacillus* decreased. These changes were reversed after Sivelestat treatment, and the microbial status was restored to resemble that of healthy rats. 2) Sivelestat altered the abundance of 11 metabolites and mainly regulated pathways including phenylalanine, tyrosine, and tryptophan biosynthesis and tyrosine metabolism. 3) Almost all Sivelestat-regulated microbes were associated with differential metabolites (*P* < 0.05), such as *Lactobacillu*s and some amino acids, suggesting that altered metabolites after Sivelestat administration were highly attributed to its effect on the microbiome.

The gut microbiota plays a crucial role in maintaining host immune homeostasis and has been proved to be associated with several diseases, including sepsis (Dickson, 2016; Dickson et al., 2016; Budden et al., 2017; Schluter et al., 2020). Sepsis can affect the gut microbiome and lead to epithelial barrier disruption and the translocation of gut bacteria, which may lead to organ failure (Zaborin et al., 2014; Haak and Wiersinga, 2017; Adelman et al., 2020). Previous studies have shown that neutrophil recruitment was directly related to alterations in the gut microbiota during infection through the release of NE-associated serine proteases leading to intestinal epithelial ligand protein degradation and altered intestinal barrier permeability, and intestinal flora balance (Ginzberg et al., 2001; Chen et al., 2021). Inhibition of NE could theoretically alleviate the disruption of gut homeostasis and have a beneficial impact.

Chen et al. showed that another NE inhibitor (MPH-966) improved the gut flora in a chemotherapy-induced intestinal mucositis model by regulating bacterial community diversity, bacterial abundance, and the *Firmicutes*/*Bacteroidetes* (F/B) ratio (Chen et al., 2021). Similarly, our study showed that the NE inhibitor Sivelestat could also restore the disordered gut microbiome. However, unlike Chen et al., who observed a decrease in the F/B ratio after MPH-966 treatment, we found that the F/B ratio increased after Sivelestat administration. This may be due to the differences in disease-induced alterations in the gut flora, with a reduced F/B in sepsis and an elevated F/B in intestinal mucositis. In addition, our study identified 36 differential OTUs between the Sive and CLP groups (*P*<0.05), suggesting that their corresponding bacteria may be mediators of the therapeutic effect of Sivelestat during sepsis. Most OTUs belonged to *Lactobacillius* in taxonomy, which were enriched in the SC and Sive groups but reduced in the CLP group. As the most widely used probiotics, *Lactobacillius* directly contribute to the nutritional, immunomodulatory, and stress resistance benefits for the host (Sengupta et al., 2013; Zhang et al., 2018), which could maintain or restore gut epithelial cell membrane integrity and attenuate sepsis-induced cell damage. *Lactobacilli* also have microbial regulatory effects that maintain the integrity of the epithelial barrier and inhibit the invasion and fixation of pathogenic bacteria such as *Escherichia-Shigella (* Yoda et al., 2014; Kozakova et al., 2016). Therefore, the Sivelestat-induced enrichment of *Lactobacillius* could help to synergize with Sivelestat to jointly promote microbial homeostasis. We also observed a reduction in *Gammaproteobacteria* abundance after Sivelestat treatment. *Gammaproteobacteria* is a class that contains many common gram-negative “pathogens”, and their proliferation could increase in an inflammatory environment (Huffnagle et al., 2017; Li et al., 2020), which was consistent with our results. Moreover, previous studies have shown that *Gammaproteobacteria* could interact with immune cells leading to the production of pro-inflammatory mediators (Huffnagle et al., 2017). Interestingly, Sivelestat administration significantly reduced the abundance of *Gammaproteobacteria*, which could potentially break this inflammatory cycle and have a therapeutic effect on sepsis. Our microbiome analysis showed that Sivelestat improved the altered gut microbiota of septic rats, which had not been reported in previous mechanistic studies of Sivelestat.

Emerging evidence has shown that microorganisms could regulate the normal development and function of the immune system and maintain immune homeostasis through their metabolites (Dang and Marsland, 2019; Fan and Pedersen, 2021). For example, short-chain fatty acids (SCFAs), key immune system regulators, are produced by the intestinal flora and can enter the peripheral circulation and bone marrow to influence immune cell activity (Martin-Gallausiaux et al., 2021). We found that Sivelestat significantly increased the levels of 11 metabolites in septic rats, most of which were reduced in the CLP group compared with the SC group, suggesting that Sivelestat could restore these metabolites to levels similar to those of healthy rats. We also observed that the abundance of four differential metabolites was low in both the SC and CLP groups but increased after Sivelestat administration. Among these, cortisone, which has anti-inflammatory effects, was the most abundant differential metabolite. The latest Surviving Sepsis Campaign (SSC) guidelines recommend the use of corticosteroids in septic shock patients (moderate quality of evidence), and our latest meta-analysis also showed that corticosteroids improved intensive care unit and in-hospital survival (Evans et al., 2021; Liang et al., 2021), supporting the beneficial effects of corticosteroids in sepsis. Further, 6-aminopenicillanic acid was the precursor of all semi-synthetic penicillin and could be introduced into different side chains to produce various antibiotics (Rolinson and Geddes, 2007). Moreover, oleoyl glycine and shikimic acid have been shown to have anti-oxidative and anti-inflammatory effects (Sun et al., 2016; Rom et al., 2020). The altered abundance of these metabolites may be one of the reasons for the significantly improved survival of septic rats treated with Sivelestat (*P*<0.05). However, the mechanism of Sivelestat-induced metabolite abundance changes and the deeper immunomodulatory mechanisms of these metabolites remain to be investigated. Our integrated microbial-metabolite analysis suggested that this effect may be related to the Sivelestat-induced alternations in the gut flora of septic rats.

This study has several limitations, including the small sample size. Also, changes in NE levels between the groups were not monitored. Besides, we did not detect common gut metabolites such as SCFAs due to the limitation of liquid-phase non targeted metabolomics. For the future directions, it would be interesting to evaluate the impact of Sivelestat on the short-term and long-term prognosis of sepsis in larger sample size. And more detailed molecular biology analyses such as evaluating the effect of Sivelestat on NE and SCFAs will be necessary for the clinical translation of Sivelestat as a treatment for sepsis.

## Conclusion

This was the first study to investigate the effect of Sivelestat on the gut microbiome and on metabolic profiles of septic rats, which could provide new approaches for sepsis treatment.

## Data Availability Statement

The datasets presented in this study can be found in online repositories. The name of the repository and accession number can be found below: NCBI; PRJNA802277.

## Ethics Statement

The animal study was reviewed and approved by the Animal Care and Use Committee of the Zhengzhou University.

## Author Contributions

All the authors contributed substantially to the work presented in this article. TS and XD conceived of the study. YS, YC, and HoL contributed to the data interpretation. YS, YC, DW, HuL, SL, XZ, and HW contributed to the study protocol. YS contributed to the writing of the article and XD, and TS revised the article. All authors have approved the final and submitted version of the manuscript.

## Funding

This study was supported by Grant of NSFC-Henan Joint Foundation of China (Grant No. U2004110), the National Natural Science Foundation of China (Grant No. 82172129); the 2021 youth talent promotion project in Henan Province (Grant No.2021HYTP053), 2021 joint construction project of Henan Medical Science and technology breakthrough plan (Grant No. LHGJ20210299).

## Conflict of Interest

The authors declare that the research was conducted in the absence of any commercial or financial relationships that could be construed as a potential conflict of interest.

## Publisher’s Note

All claims expressed in this article are solely those of the authors and do not necessarily represent those of their affiliated organizations, or those of the publisher, the editors and the reviewers. Any product that may be evaluated in this article, or claim that may be made by its manufacturer, is not guaranteed or endorsed by the publisher.

## Supplementary Material

Supplementary Table 1 Alpha diversity index of gut microbiota in the experimental groups.

Supplementary Figure 1
**(A)** Rank Abundance Curve reflecting the richness and evenness of species. **(B)** Species Accumulation Boxplot showing that the sample size is adequate. **(C)** ANOVA analysis based on weighted unifrac distance. *****Compared with the SC group; **^#^** compared with the CLP group; **^*^** ^,^ **^#^** *P*<0.05; **^**^** ^,^ **^##^** *P*<0.01; **^***^** ^,^ **^###^** *P*<0.001. **(D)** Unweighted Pair-group Method with Arithmetic Mean (UPGMA) clustering tree based on weighted unifrac distance.

Supplementary Figure 2 The phylogenetic tree of the top 100 species at the genus level. The colors of the branches and sectors indicate the corresponding phylum, and the stacked bars on the outside of the fan ring indicate the abundance distribution of the genus in the different groups.

Supplementary Figure 3 The total ion chromatograms of the SC **(A, B)**, CLP **(C, D)**, and Sive **(E, F)** groups. **(A, C, E)** Negative ion mode; **(B, D, F)** Positive ion mode.

Supplementary Figure 4 Metabolites and microbial-metabolite correlation analysis. **(A)** Heatmap of the correlation analysis for the Sive and CLP groups. **(B)** Microbial-metabolite correlation analysis within the CLP group. **(C)** Microbial-metabolite correlation analysis within the Sive group. **^*^** *P*<0.05.

Supplementary Figure 5 Heatmap of the correlation analysis of differential bacterial genera and metabolites between the Sive and CLP groups.
