# Supplementary material for: Positive Effects of Neutrophil Elastase Inhibitor (Sivelestat) on Gut Microbiome and Metabolite Profiles of Septic Rats
Source: Front Cell Infect Microbiol. 2022 Mar 15;12:818391. doi: 10.3389/fcimb.2022.818391 (PMC8965314; doi:10.3389/fcimb.2022.818391)
Supplement: Supplementary Table 1 — Alpha diversity index of gut microbiota in the experimental groups. [file Table_1.docx]

Supplement Table 1. Alpha diversity index of gut microbiota in the experimental groups.

| Group | observed_species | shannon | simpson | chao1 | ACE | goods_coverage | PD_whole_tree |
| --- | --- | --- | --- | --- | --- | --- | --- |
| SC | 479.8±60.7 | 5.4±0.5 | 0.93±0.03 | 508.2±68.5 | 514.9±67.6 | ＞99% | 41.5±6.1 |
| CLP | 515.8±86.9 | 5.6±0.9 | 0.89±0.09 | 525.0±98.7 | 557.9±96.4 | ＞99% | 41.6±5.5 |
| SIVE | 460.2±42.6 | 5.5±0.4 | 0.84±0.02 | 488.3±46.4 | 491.6±40.7 | ＞99% | 37.8±6.0 |
| *P* value (ANOVA) | 0.452 | 0.881 | 0.337 | 0.743 | 0.389 | 0.138 | 0.520 |
