# Supplementary figures and images for: Positive Effects of Neutrophil Elastase Inhibitor (Sivelestat) on Gut Microbiome and Metabolite Profiles of Septic Rats
Source: Front Cell Infect Microbiol. 2022 Mar 15;12:818391. doi: 10.3389/fcimb.2022.818391 (PMC8965314; doi:10.3389/fcimb.2022.818391)

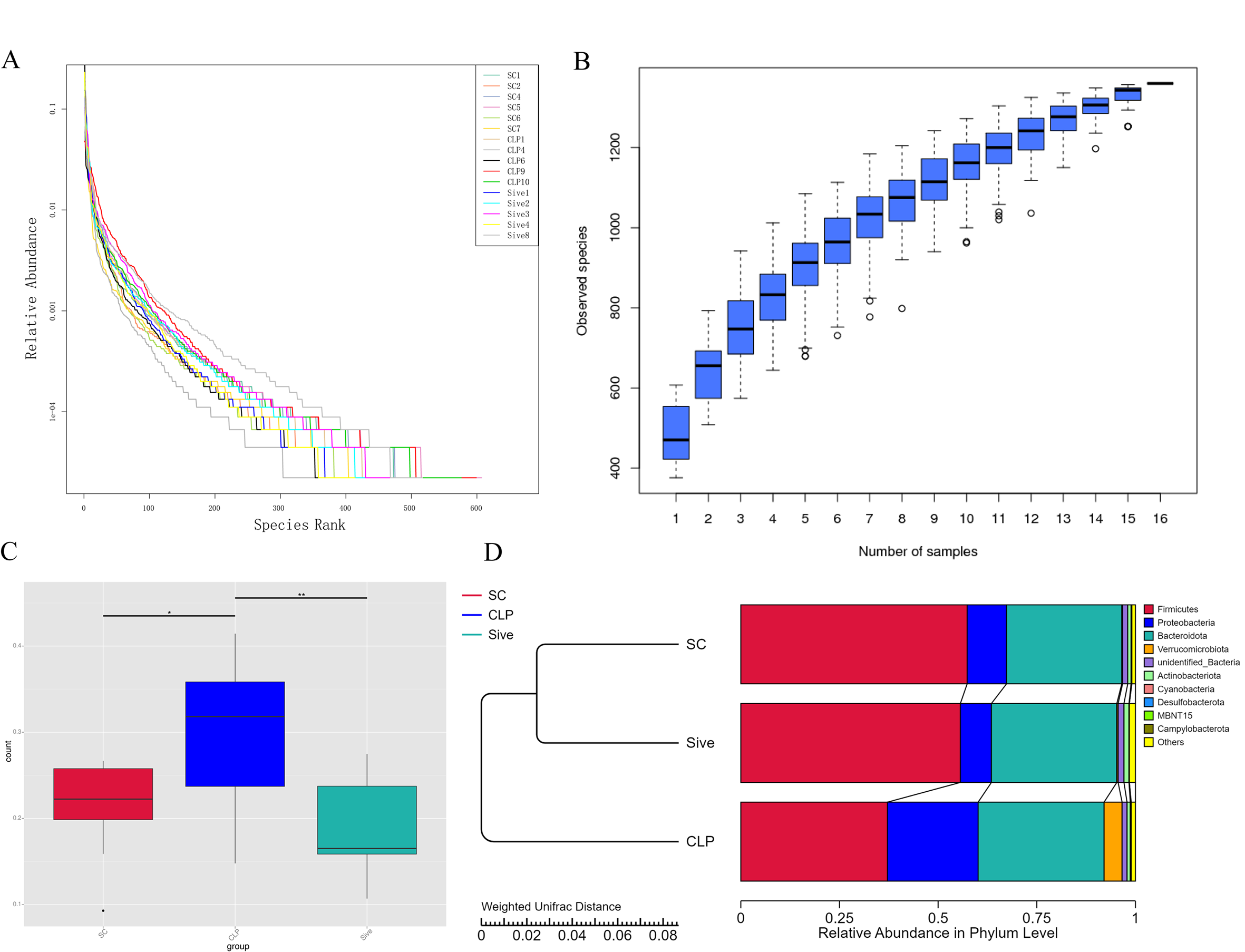

Supplement: Supplementary Figure 1 — (A) Rank Abundance Curve reflecting the richness and evenness of species. (B) Species Accumulation Boxplot showing that the sample size is adequate. (C) ANOVA analysis based on weighted unifrac distance. *Compared with the SC group; # compared with the CLP group; * , # P<0.05; ** , ## P<0.01; *** , ### P<0.001. (D) Unweighted Pair-group Method with Arithmetic Mean (UPGMA) clustering tree based on weighted unifrac distance. [file Image_1.tif]

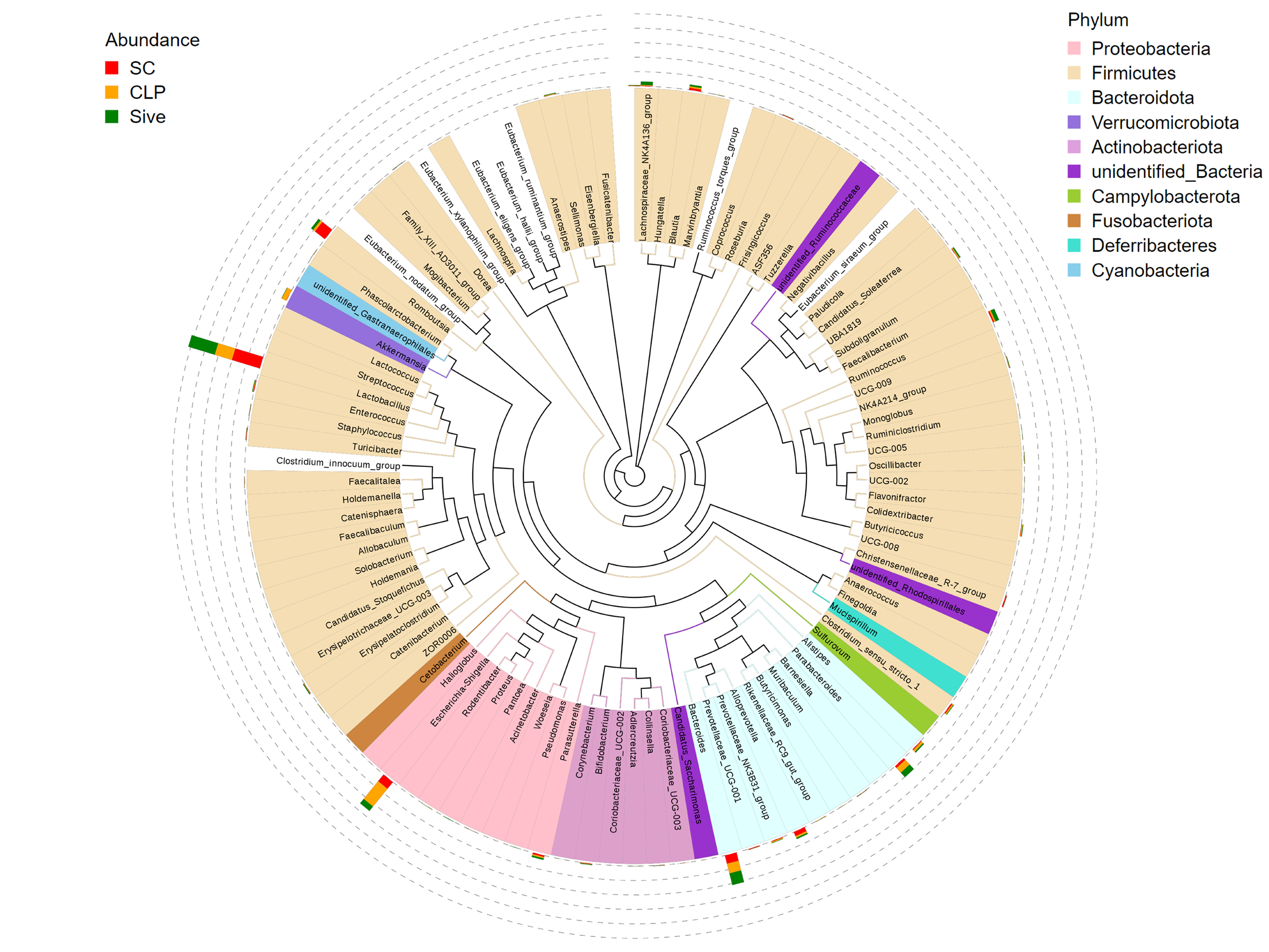

Supplement: Supplementary Figure 2 — The phylogenetic tree of the top 100 species at the genus level. The colors of the branches and sectors indicate the corresponding phylum, and the stacked bars on the outside of the fan ring indicate the abundance distribution of the genus in the different groups. [file Image_2.tif]

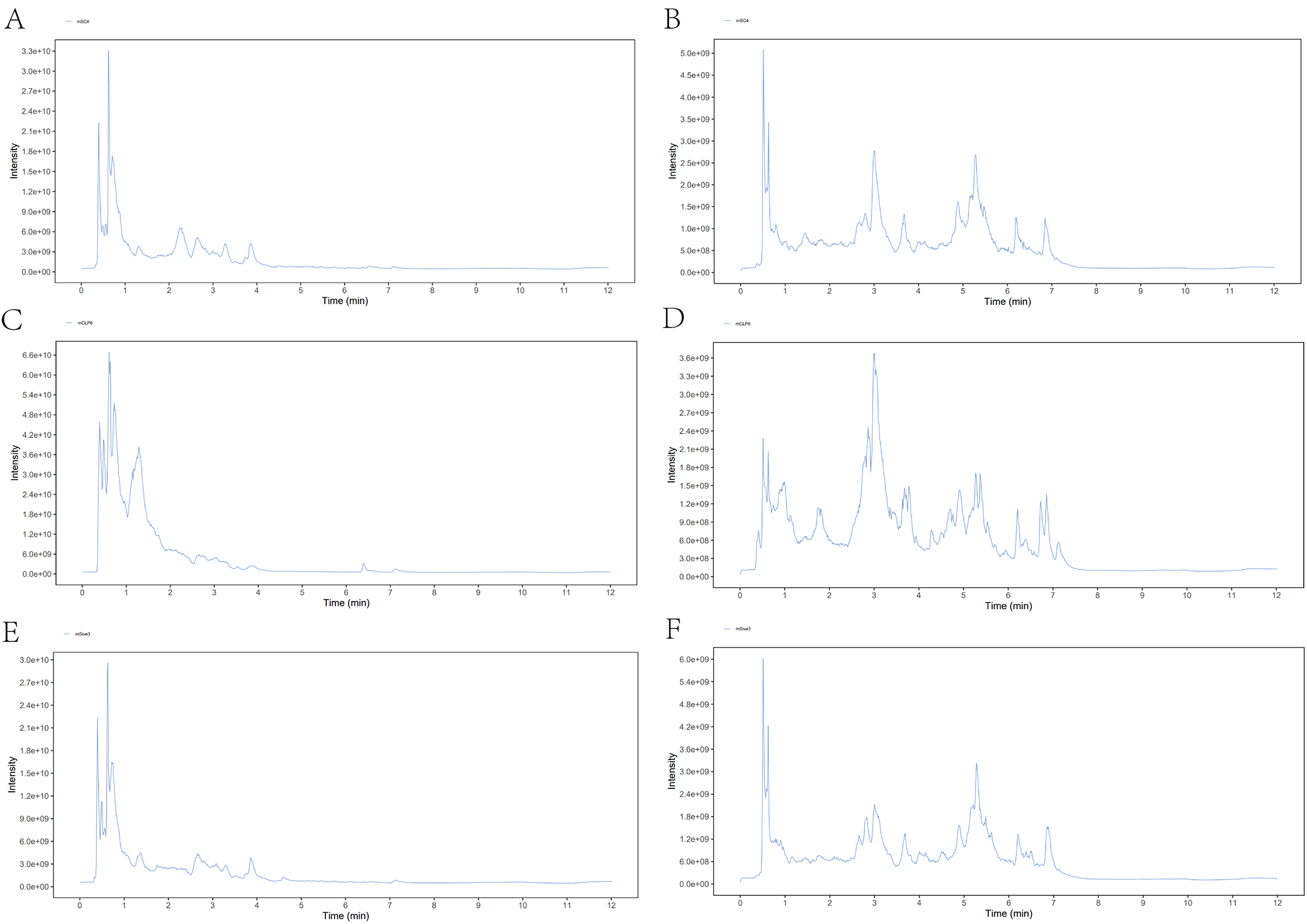

Supplement: Supplementary Figure 3 — The total ion chromatograms of the SC (A, B), CLP (C, D), and Sive (E, F) groups. (A, C, E) Negative ion mode; (B, D, F) Positive ion mode. [file Image_3.tif]

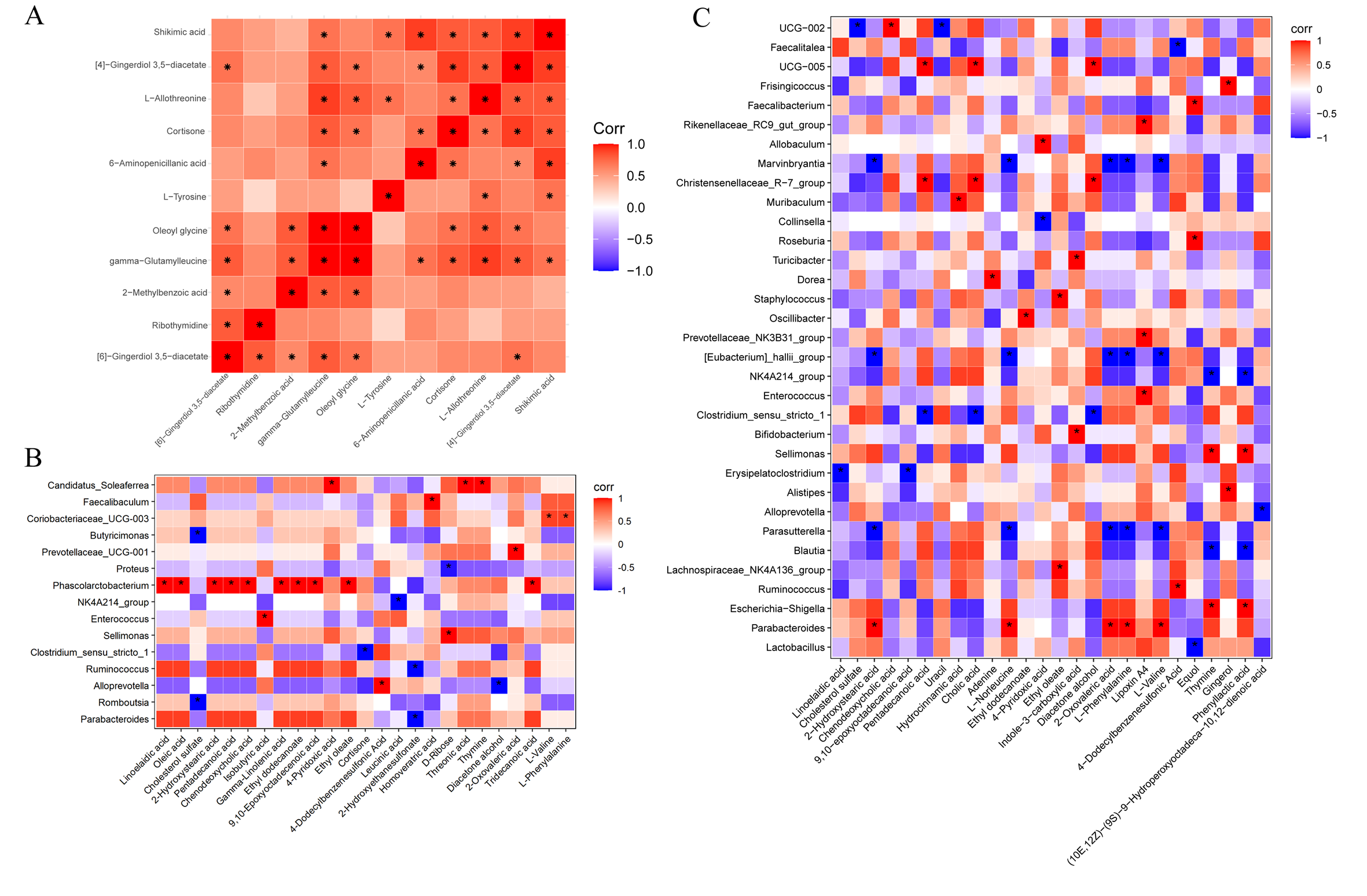

Supplement: Supplementary Figure 4 — Metabolites and microbial-metabolite correlation analysis. (A) Heatmap of the correlation analysis for the Sive and CLP groups. (B) Microbial-metabolite correlation analysis within the CLP group. (C) Microbial-metabolite correlation analysis within the Sive group. * P<0.05. [file Image_4.tif]

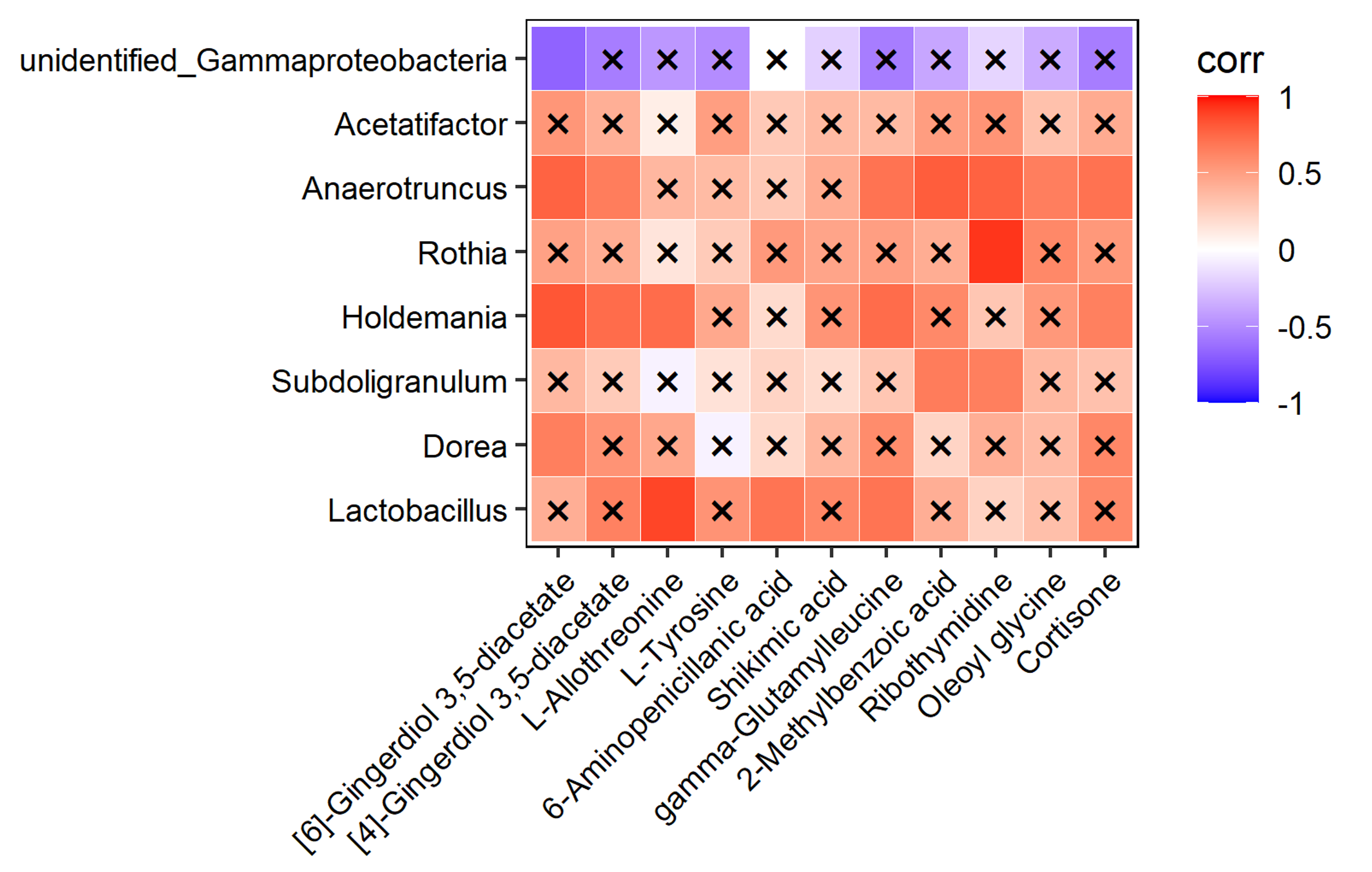

Supplement: Supplementary Figure 5 — Heatmap of the correlation analysis of differential bacterial genera and metabolites between the Sive and CLP groups. [file Image_5.tif]
